# Mitochondrial Quality Control in Hepatocellular Carcinoma

**DOI:** 10.3389/fonc.2021.713721

**Published:** 2021-09-13

**Authors:** Jinda Bian, Dan Zhang, Yicun Wang, Hanjiao Qin, Wei Yang, Ranji Cui, Jiyao Sheng

**Affiliations:** ^1^Department of Hepatobiliary and Pancreatic Surgery, The Second Hospital of Jilin University, Changchun, China; ^2^Jilin Provincial Key Laboratory on Molecular and Chemical Genetic, The Second Hospital of Jilin University, Changchun, China; ^3^Department of Radiotherapy, The Second Hospital of Jilin University, Changchun, China

**Keywords:** mitochondrial quality control, hepatocellular carcinoma, tumorigenesis, tumor progression, chemoresistance

## Abstract

Mitochondria participate in the progression of hepatocellular carcinoma (HCC) by modifying processes including but not limited to redox homeostasis, metabolism, and the cell death pathway. These processes depend on the health status of the mitochondria. Quality control processes in mitochondria can repair or eliminate “unhealthy mitochondria” at the molecular, organelle, or cellular level and form an efficient integrated network that plays an important role in HCC tumorigenesis, patient survival, and tumor progression. Here, we review the influence of mitochondria on the biological behavior of HCC. Based on this information, we further highlight the need for determining the role and mechanism of interaction between different levels of mitochondrial quality control in regulating HCC occurrence and progression as well as resistance development. This information may lead to the development of precision medicine approaches against targets involved in various mitochondrial quality control-related pathways.

## 1 Introduction

Hepatocellular carcinoma (HCC) accounts for approximately 75% of primary liver cancers Current therapies for HCC, including surgery, chemotherapy, and targeted therapy, are not effective for treating advanced HCC because they do not prevent the proliferation, metastasis, invasion, angiogenesis, and chemoresistance of this cancer (1–5); therefore, the 5-year relative survival rate of patients with HCC remains at only 18% (6). It has been reported that mitochondria can participate in the progression of HCC by modifying redox homeostasis (7), metabolism (8, 9), and the cell death pathway (10); these complex regulatory networks play important roles in tumorigenesis, patient survival, and tumor progression and depend on the mitochondrial health status. Cells can repair or eliminate “unhealthy mitochondria” *via* mitochondrial quality control to balance mitochondrial homeostasis. Several studies have focused on the influence of mitochondrial quality control on the maintenance of cell viability and chemoresistance by evaluating the metabolic pathways specific to liver cells. These studies identified key mitochondrial quality control molecules as new targets for liver cancer treatment. Here, we review the effects of mitochondrial quality control-related pathways and molecules that maintain cell survival on the efficacy of liver cancer therapy and provide new candidates for mitochondria-targeted liver cancer treatments.

## 2 Mitochondria Regulate the Progression and Death of Hepatocellular Carcinoma

The mitochondrial matrix is surrounded by two lipid membranes: the outer mitochondrial membrane and inner mitochondrial membrane (IMM). Various proteins and protein complexes are present on the mitochondrial membrane. Mitochondria function to supply energy to cells through oxidative phosphorylation, such as by producing ATP through the electron transport chain (ETC) and by using oxygen. This process is accompanied by the generation of reactive oxygen species (ROS) (11). Mitochondria are abundant in tumor cells, although aerobic glycolysis is the main mode of energy metabolism in these cells. This high-speed, low-efficiency mode of metabolism is known as the Warburg effect, and it allows cells to grow rapidly and promotes the tumor formation, proliferation, and progression (12, 13). Moreover, mitochondria in tumor cells maintain complete metabolic functions and play important roles in tumor metabolism. Studies have shown that phenformin inhibits the mitochondrial respiratory chain complex I to suppress the proliferation of liver cancer cells (14). Inhibiting the expression of mitochondrial ATP synthase subunit e restricts cell proliferation (15). These findings also indicate that mitochondria affect tumor cell metabolism and alter the biological behavior of a tumor. Additionally, when tumor cells respond to external stress stimulation, they maintain their survival through mitochondria-mediated metabolic reprogramming.

The liver is a metabolic factory in humans. Therefore, mitochondria are more abundant in liver cells than in other types of cells (16), suggesting that mitochondria play an important role in the occurrence and development of liver cancer. Excessive ROS production by mitochondria in hepatocytes can induce tumorigenesis. Rostkowska et al. found that N-nitrosodiethylamine induces excessive production of free radicals that disrupt protein folding and increase ATP demand in hepatocyte mitochondria. These effects indirectly increase mitochondrial oxidative phosphorylation and ROS accumulation, eventually leading to liver carcinogenesis (17–21). Preventive elimination of mitochondria-derived ROS (mtROS) has been suggested to inhibit the occurrence of HCC (22). In tumor cells, moderate levels of mtROS can stimulate the expression of nuclear genes involved in angiogenesis and oxygen supply, glycolysis, anti-oxidation, cell proliferation, epithelial-mesenchymal transition, anti-apoptosis, invasion, and metastasis through mitochondrial retrograde signaling pathways that help cells adapt to new metabolic requirements (23). For example, the production of low levels of mtROS can induce the proliferation of HCC cells through phosphoinositide 3-kinase-mediated phosphorylation of Akt (24). However, excessive levels of ROS can also have a tumor-killing effect. Several previous studies, including a study conducted by our group, have revealed that cisplatin-based chemotherapy drugs destroy liver cancer cells by inducing an increase in mtROS levels and inhibiting HCC proliferation. The increase in ROS levels induced by lenvatinib can promote apoptosis in liver cancer cells through the ATM and eIF2α signaling pathway (25, 26).

Mitochondria are also central organelles that regulate cell death such as apoptosis, necrosis, ferroptosis, pyroptosis, and other cell death processes. An increased expression of anti-apoptotic members of the Bcl-2 protein family (Bcl-XL and Mcl-1), which are localized to the mitochondria in HCC cells, can induce resistance to regorafenib and sorafenib (27, 28). Resistance to apoptosis also plays a role in the chemoresistance mechanisms of liver cancer cells. For example, an increased expression of mitochondrial phosphoglycerate mutase/protein phosphatase in liver cancer cells enhances chemoresistance to 5-fluorouracil by increasing the stability of Bcl-XL, and inhibiting the expression of Bax and cytochrome c, which eventually inhibit the apoptotic signaling pathway (29). A study suggested that HCC can resist apoptosis by overexpressing mitochondrial uncoupling protein to decrease mitochondrial membranes permeability (30). In addition to apoptosis, mitochondria are involved in ferroptosis and necrosis. Genes related to mitochondrial iron and fatty acid metabolism (*RPL8*, *IREB2*, *ATP5G3*, *CS*, *TTC35*, and *ACSF35*) can provide the required lipid precursors for mitochondrial ferroptosis and excess iron to induce ROS. Levels of ROS that exceed the antioxidant capacity of cells can cause oxidative stress, which directly or indirectly damages various macromolecules, such as proteins, nucleic acids, and lipids (31), and triggers the release of lipid peroxides from the mitochondria that leads to cell ferroptosis (32). Moreover, overexpression of mitochondrial CISD1 (CDGSH iron-sulfur domain 1) can inhibit ferroptosis in HCC cells by regulating membrane lipid peroxidation and mitochondrial iron uptake (33). These findings suggest that mitochondria play a role in regulating iron-induced death in HCC cells. To induce necroptosis, mitochondrial ROS activate autophosphorylation of receptor-interacting protein kinase 1, leading to the recruitment of RIPK3 to further promote the production of ROS. This forms a positive feedback loop that promotes necroptosis (11, 34–37). Recent studies have shown that in HepG2 cells with low levels of GSNOR, inhibition of the mitochondrial respiratory chain complex II expression leads to necroptosis of the PARP1/receptor-interacting protein kinase 1 pathway and suppresses cell growth (38). However, mitochondrial glutathione in HepG2 cells increases the tolerance of cells to azathioprine by protecting cytochrome C and inhibiting necroptosis *via* the JNK pathway (39). Pyroptosis is a process induced by inflammasomes, as an immune response to bacteria, pathogens, or their endotoxins (40). One of the main mechanisms is gasdermin E(GSDME)-dependent pyroptosis. A recent study has found that the mitochondrial apoptotic protein caspase3/7 can be activated by GSDME and then participate in cell apoptosis, additionally, mitochondrial endogenous apoptotic protein caspase3/9 can also induce GSDME-dependent pyroptosis (41), suggesting that mitochondria play an important role in the crosstalk between apoptosis and pyroptosis. NLRP3 as a kind of inflammasome was found to activate caspase-1, which results in pyroptosis activation. Yu et al. established a HCC cell model of Non-alcoholic steatohepatitis using double stimulation with palmitic acid and lipopolysaccharide, which caused mitochondrial dysfunction and ROS generation. ROS overproduction activated inflammasome by binding to NLRP3, and then initiate pyroptosis. But PINK/Parkin-dependent mitophagy induced by liraglutide could eliminate damaged mitochondria and inhibit pyropotosis (42).

**Figure 1:**
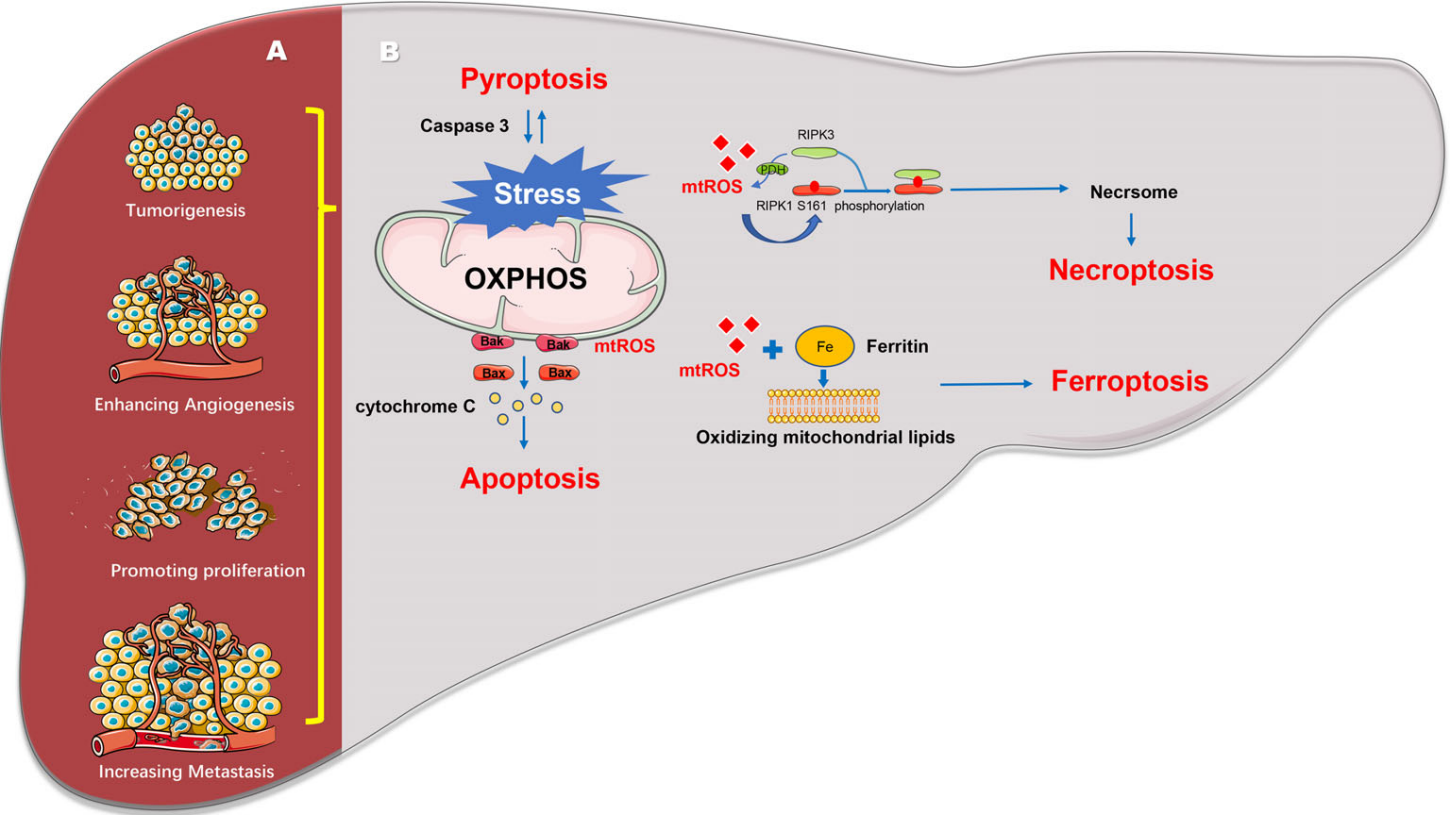
Mitochondrial regulates the progression and death of hepatocellular carcinoma. **(A)** Mitochondria can reverse regulate the expression of nuclear genes to induce cell tumorigenesis, angiogenesis, cell proliferation, invasion, and metastasis. **(B)** Mitochondria can regulate various cell death pathways including apoptosis, necroptosis, pyroptosis and ferroptosis and so on.

In summary, stable energy and metabolism, redox homeostasis of mitochondria, and regulation of liver cancer cell death influence the tumorigenesis and progression of HCC (**Figure 1**). The maintenance of mitochondrial homeostasis is the basis of influence of development. Several studies have shown that HCC can maintain mitochondrial homeostasis through mitochondrial quality control, enabling the proliferation, invasion, chemotherapy resistance, and survival of HCC cells. Mitochondrial quality control changes occur at the molecular, organelle, and cellular levels to maintain mitochondrial function or eliminate damaged mitochondria to ensure mitochondrial and cellular homeostasis (21, 43).

## 3 Molecular Mechanisms Involved in the Quality Control of Mitochondria

In response to external nutrient deficiency, hypoxia, oxidative stress, abnormal metabolite accumulation, viruses, and other pathophysiological factors, mitochondria can stimulate the mitochondrial quality control system to ensure that a sufficient number of functional mitochondria are present to meet the cellular needs in case of cell damage (43, 44). Proteins in the mitochondria are encoded by nuclear and mitochondrial genes. Moreover, mitochondrial quality control is an extremely complicated system involving coordinated protein synthesis and mitochondrial import of nuclear gene-encoded proteins.

Most HCC patients are accompanied by viral hepatitis, alcoholic liver disease, or non-alcoholic fatty liver disease (NAFLD), which causes the microenvironment of HCC to be different from that of other tumors. These factors also affect the mitochondrial status and mitochondrial quality control. Studies have shown that HCC cells can induce the down-regulation of MFN2 and the recruitment of DRP1 to mitochondria after HCV infection, causing mitochondrial fission and subsequent mitophagy. These changes in mitochondrial quality control can help reduce HCC cell apoptosis and may lead to persistent HCV infection (45). A similar phenomenon was also found in HBV-infected HCC cells. HBV can induce mitochondrial translocation of Drp1. In addition, HBV upregulates the expression of genes *Parkin, PINK1* and *LC3B* and induces Parkin recruitment to mitochondria and MFN2 degradation by ubiquitination. Mitochondrial fission and mitophagy also promote the survival of HCC cells and may promote the continued existence of HBV (46). In an experiment using oleic acid to treat HCC cells to build NAFLD model, it was found that oleic acid induced NAFLD can induce mitophagy in HCC cells (47). In addition, alcohol can also damage the mitochondria of HCC cells and induce mitochondrial quality control (48). Above all, compared with other tumor cells, HCC cells may need to adapt to the unique tumor microenvironment through mitochondrial quality control for maintaining its own survival.

### 3.1 Transport of Mitochondrial Proteins and Mitochondrial Unfolded Protein Response in HCC

**Figure 2:**
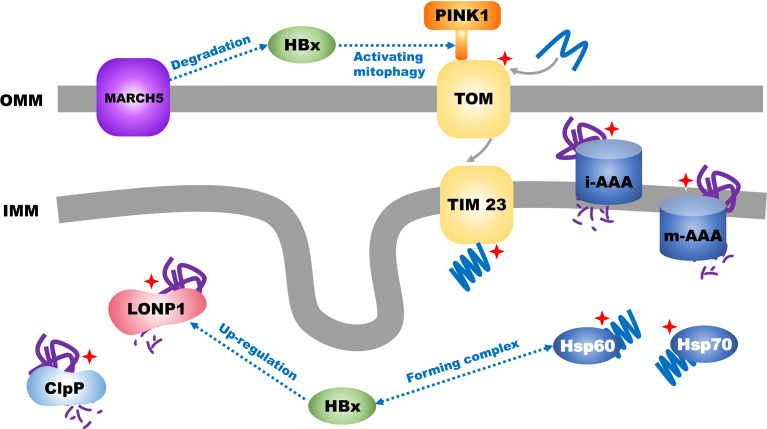
Transport of mitochondrial proteins and mitochondrial unfolded protein response in HCC. Mitochondrial unfolded protein response is labelled with red star.

The nucleus encodes most mitochondrial proteins that undergo unfolding and refolding to enter the mitochondria (43, 49). Mitochondria produce ROS during energy metabolism, leading to damage to the mitochondrial DNA (mtDNA). These factors eventually interfere with protein folding. Therefore, to ensure mitochondrial homeostasis, cells must degrade abnormal (misfolded or unfolded) proteins *via* the mitochondrial unfolded protein response (mtUPR) (**Figure 2**) (43). This method of degradation or refoldation separately depends on mitochondrial proteases and chaperone proteins, such as mitochondrial heat shock protein 60, TNF receptor-associated protein 1 (TRAP1), and mortalin (HSPA9) as well as the ubiquitin-proteasome system for mitochondrial quality control at the molecular level (50). In the mitochondrial matrix, misfolded or unfolded proteins are degraded by mitochondrial proteases (LON and mtClpP) or refold by mitochondrial protein chaperones (Hsp60/Hsp70/Hsp100). Misfolded or unfolded proteins in the inner mitochondrial membrane, particularly unassembled subunits of the respiratory chain complex, are mainly degraded by two membrane-integrated protease systems (i-AAA/m-AAA) and two AAAs (ATPases associated with diverse cellular activities) that belong to the AAA+ enzyme family (51, 52). In the outer mitochondrial membrane, misfolded or unfolded proteins are ubiquitinated by the ubiquitin-proteasome system in the cytosol and then degraded (53, 54).

In mammals, the mtUPR is triggered by an integrated stress response caused by misfolded proteins in the mitochondria. The integrated stress response promotes overexpression of ATF5 by phosphorylating the translation initiation factor eIF2α (55, 56). Under normal conditions, ATF5 is introduced into the mitochondria by its own mitochondrial targeting sequence for degradation; however, under mitochondrial-nucleoprotein imbalance conditions, the nuclear localization signal of ATF5 binds to the nuclear mtUPR element. This initiates the transcription of genes related to the mtUPR, including HSP60, HSP10, and LONP1. These transcripts are translated and introduced into the mitochondria (anterograde signals). Inside the mitochondria, molecular chaperones, such as Hsp60 and Hsp10, co-fold damaged proteins, and mitochondrial proteases, such as LONP1, cut and degrade the irreparable proteins to maintain mitochondrial protein homeostasis (57).

Approximately 40% of HCCs are caused by hepatitis B virus (HBV) infection (58). HBV can promote the progression and worsen the prognosis of HCC (59) through the expression of the open reading frame of HBV X protein (HBx). Xu et al. found that HBx could promote an increase in the expression of ATF5 through the HPIP/AKT/ERK/FOXO4 pathway (60); another study showed that HBx could form a complex with the mtUPR molecular chaperones HSP60 and HSP70 (61) which facilitate HBx-induced cell apoptosis (62). Furthermore, HBx can promote the mtUPR. Studies have found that HBx can induce the translocation of LONP1 from the cytoplasm to the mitochondria and activate the LONP1/PINK1 pathway to promote mitochondrial autophagy and eventually inhibit the mitochondrial apoptotic pathway (63). However, HBx in the mitochondria is also regulated by the ubiquitination system of the outer mitochondrial membrane in the mtUPR; the outer mitochondrial membrane mitochondrial ubiquitin ligase (MARCH5) mediates the degradation of HBx *via* the ubiquitin proteasome and inhibits ROS generation, stabilizes the quality of mitochondria, reduces inflammation caused by HBx, and inhibits the initiation of HCC. Western blotting and mRNA expression analyses of tumor tissues from patients with HCC have suggested that the expression of MARCH5 gradually decreases with increasing tumor grades (G1–G4), and Kaplan-Meier analysis has shown that high MARCH5 expression is associated with prolonged survival in patients with HCC (64). Efavirenz (EFV) is the most widely used non-nucleoside reverse transcriptase inhibitor and has been shown to cause liver damage by affecting mitochondrial homeostasis in hepatocytes. Nadezda et al. found that in Hep3B cells, EFV could induce endoplasmic reticulum stress, which is mediated by the mitochondria. However, the harmful effects of EFV are markedly reduced in cells lacking functional mitochondria (65). Another study has shown that although EFV can reduce the content of LON in the mitochondria in Hep3B cells, it can increase its content in the outer membrane of mitochondria and mitochondria-associated endoplasmic reticulum membranes, and thus, it may lead to mitochondrial/endoplasmic reticulum stress in HCC cells (66).

In addition to maintaining the homeostasis of mitochondrial proteins, studies have found that the degradation of the mitochondrial chaperone, TRAP1, induced by *S*-nitrosylation, leads to increased succinate dehydrogenase activity and inhibition of mitochondrial complex IV expression. Increased succinate dehydrogenase activity sensitizes HepG2 cells to α-tocopheryl succinate (α-TOS), which is a succinate dehydrogenase inhibitor, and oxidative stress caused by the mitochondrial targeting agent mitocans. Additionally, α-TOS can inhibit the proliferation of HCC cells by degrading TRAP1 and inducing necroptosis (38).

Proper transport of mitochondrial proteins ensures both mitochondrial protein and nucleic acid homeostasis. For example, the mitochondrial transport protein mia40 is essential for transporting purine/pyrimidine endonuclease 1, which repairs mtDNA. Moreover, as cell proliferation is accelerated in the early stage of HCC, high expression of mia40 promotes purine/pyrimidine endonuclease 1 mitochondrial translocation, which is beneficial for maintaining mitochondrial homeostasis during the rapid cell proliferation stage (67).

Few studies have focused on the mitochondrial proteases involved in the mtUPR in the mitochondrial matrix and intima in HCC cells, with most studies investigating the diagnosis, evaluation of the therapeutic effect, and prognosis of HCC (68–71). These limited studies of the mtUPR molecular chaperones and proteases have shown that mtUPR has a promotes HCC progression. Therefore, further research is needed to clarify whether these molecules affect the biological behavior of tumor cells and are related to the regulation of mitochondrial quality control.

### 3.2 Mitochondrial Quality Control at the Organelle Level in HCC

**Figure 3:**
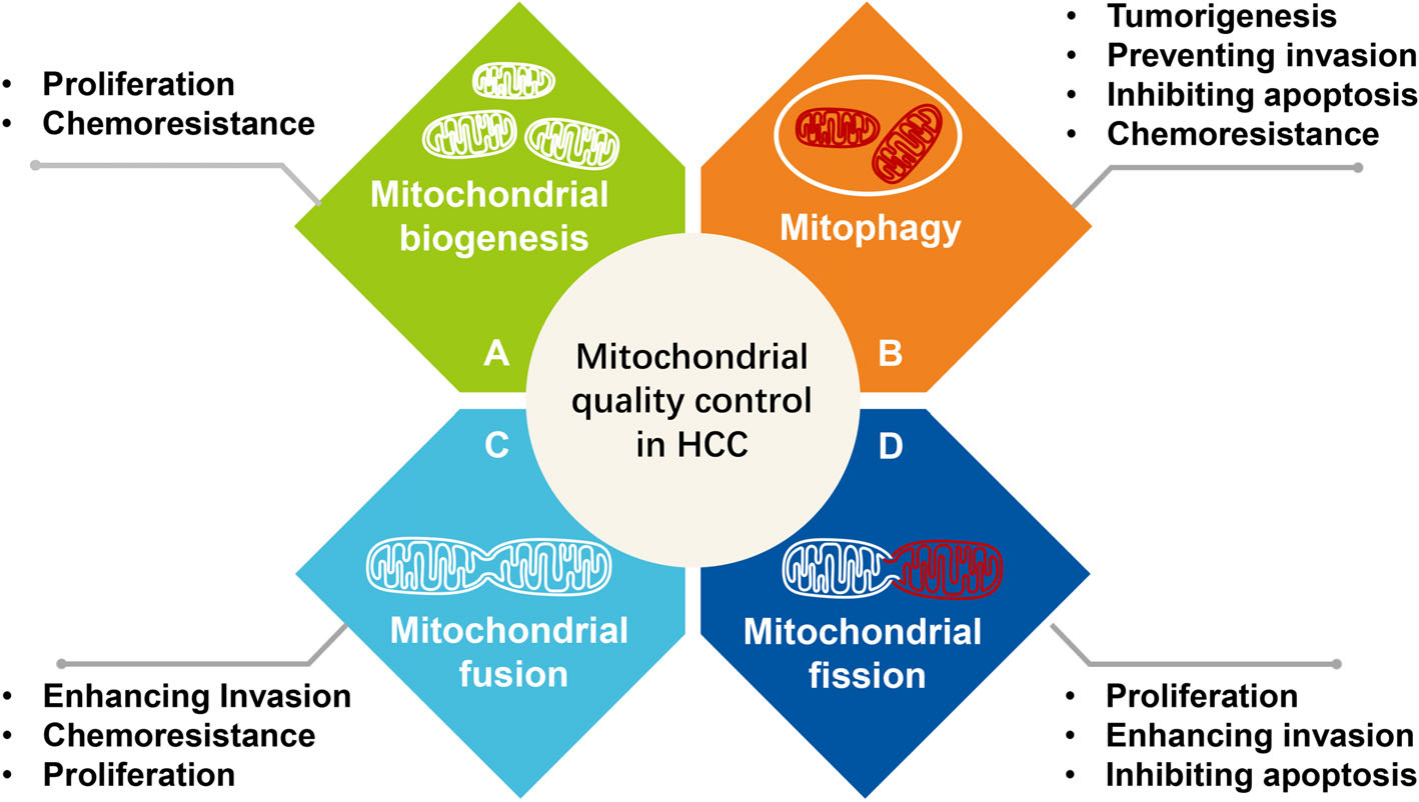
The effect of mitochondrial quality control at the organelle level on the survival and progression of hepatocellular carcinoma. **(A)** Mitochondrial biogenesis, **(B)** Mitophagy, **(C)** Mitochondrial fusion, **(D)** Mitochondrial fission.

Mitochondria are highly motile and are transported along the cytoskeleton. As the degree of stress increases, mitochondria and cells initiate mitochondrial quality control at the organelle level, which involves mitochondrial fusion, fission, mitophagy, and mitochondrial biogenesis (**Figure 3**).

Mitochondrial fission is the division of a mitochondrion into two smaller mitochondria. This division starts with the recruitment of cytoplasmic Drp1 (also known as DNM1L) by mitochondrial outer membrane proteins—mitochondrial fission 1 protein (FIS1) and mitochondrial fission factor (MFF)—following which Drp1 oligomerizes and drives scission (72, 73). The recruited Drp1 combines with mitochondrial outer membrane proteins (FIS1, MFF, MID49, and MID51) and then oligomerizes to form a band, compressing the site at which mitochondrial division occurs (74). The hydrolysis of GTP in the MID49/MID51–DRP1–GTP complex assists in shrinking of the complex, which curls into a closed loop with an inner diameter of 16 nm until the outer mitochondrial membrane shrinks (75). The cleaved mitochondria can be further transported by autophagosomes to lysosomes for degradation, a process known as mitophagy (44), making mitochondrial fission an important mode of mitophagy sorting. Under hypoxic conditions, HCC cells increase mitochondrial fission by overexpressing Drp1 and increasing expression of the mitophagy-related protein, BNIP3, to promote the mitophagy and survival of HCC cells. However, after Midiv-1 inhibits fission, hypoxia induces apoptosis *via* the mitochondrial pathway in HCC cells. This suggests that HCC cells can use mitochondrial fission as a stress-responsive mechanism to maintain cell survival (76). Other studies have shown that mitochondrial division plays a role in the separation of mitochondria with damaged proteins, membrane instability, and mutations or damaged mtDNA (77–81). Additionally, mitochondrial division markedly affects the production of ROS (82), regulation of cell proliferation (83–86), and apoptosis (87).

Mitochondrial fusion is a mechanism through which mitochondria combine. This process requires two mitochondria to be in close contact (88). Once a close connection is established, two MFN1 molecules dock in the *trans* position, possibly *via* the HR2 or GTPase domain; this association induces conformational changes that drive GTP hydrolysis by MFN1 molecules, leading to the fusion of the two outer mitochondrial membranes (73). Further, IMM fusion mediated by OPA1 depends on the inner membrane potential. However, fusion helps balance the matrix metabolite levels, regulate the distribution of mtDNA in the mitochondrial network, or help balance the levels of membrane components, such as electron transport chain complex I (77, 89–92). These components enable mitochondria to resist metabolic damage, maintain cell integrity and prevent autophagy, and improve the aerobic metabolism of cells to respond to stimuli, such as starvation and low protein synthesis (93, 94).

Mitophagy is an important mechanism of mitochondrial quality control at the organelle level. Normally PINK1 is imported into the IMM, and a lack of import induces mitophagy by stabilizing PINK1 to recruit Parkin1 in mitochondrial outer membrane (44, 95). Mitophagy involves five steps: formation of isolation membrane, elongation, closure of the isolation membrane and autophagosome formation, autophagosome–lysosome fusion, and lysosomal degradation (96). PINK1 is constitutively imported, likely *via* the TIM/TOM complex, to the inner membrane, where it is cleaved by several proteases and then proteolytically degraded (97–100). Accumulation of PINK1 on the mitochondrial surface recruits Parkin from the cytosol to the damaged mitochondria. After mitochondrial translocation, the E3 ubiquitin ligase activity of Parkin increases (101), which mediates the formation of two types of polyubiquitin chains: lysine (K) 48 linkage associated with proteasomal degradation of the substrate and lysine (K) 63 linkage associated with autophagic degradation (98). K48-mediated degradation of substrates, such as Miro and Mitofusin, inhibits mitochondrial fusion and transport, whereas K63 is recognized by ubiquitin-binding adaptors, such as p62, HDAC6, and other unknown ubiquitin-binding adaptors, that recruit damaged mitochondria to the isolation membrane by interacting with the autophagosomal protein LC3 (44). This pathway is known as PINK1/Parkin-dependent mitochondrial autophagy.

Furthermore, the mitochondrial outer membrane proteins—BNIP3, NIX, and FUNDC1—combine with the isolation membrane LC3 to participate in recruiting and extending the isolation membrane, eventually leading to the formation of autophagosomes (44, 50). BNIP3 is a Bcl-2 homology 3only protein with an LC3-binding motif known as the LC3 interaction region and binds to the isolation membrane LC3/GABARAP to promote mitophagy (102). Nix (BNIP3L), a mitochondrial outer membrane protein related to Bcl2, has an atypical BH3 domain containing a conserved LC3 interaction region; thus, it may be similar to the yeast recruiting autophagy ATG32/ATG11 receptors that target autophagosomes. FUNDC1 is present on the outer mitochondrial membrane. Under normal conditions, FUNDC1 is phosphorylated by Src kinase and CK2 at Tyr18 and Ser13, respectively, to reduce its affinity for LC3. Under hypoxic conditions or following the loss of mitochondrial potential, FUNDC1 is dephosphorylated by phosphoglycerate mutase/protein phosphatase and accumulates through the mitochondrial-endoplasmic reticulum junction, binds to the isolation membrane LC3 through the LC3 interaction region sequence, and recruits Drp1 to promote mitochondrial phagocytosis by autophagosomes (103). Autophagosome encapsulation triggers lysis in lysosomes (104–106) and maintains a healthy mitochondrial population (21). When mitophagy eliminates damaged mitochondria before caspase-dependent apoptosis is activated, mitochondrial autophagy functions as a cell survival mechanism.

An excessive reduction in the mitochondrial mass caused by persistent mitochondrial fission or mitophagy is not conducive to cell survival; these processes induce the cell to undergo apoptosis ensure mitochondrial quality. Mitochondrial biogenesis refers to the coordinated expression of genes in the nuclear and mitochondrial genome that help generate new mitochondria from existing mitochondria, which includes the synthesis of internal and external mitochondrial membranes and mitochondrial gene-encoded proteins, synthesis and entry of nuclear gene-encoded mitochondrial proteins, and mtDNA replication. Of these, peroxisome proliferation-activated receptor-γ co-activator-1α (PGC-1a) is considered the main regulator of mitochondrial biogenesis. It interacts with two key nuclear transcription factors, nuclear respiratory factor 1 and 2 (NRF1 and NRF2), and increases the expression and activity of NRF1 and NRF2 through protein interactions. NRF1 and NRF2 activate mitochondrial transcription factor A (TFAM) and bind to the promoter regions of nuclear genes encoding the five subunits of the ETC complex, thereby increasing the assembly of respiratory complexes and regulating participation in heme biosynthesis, transcription of nuclear genes encoding mitochondrial proteins, and mtDNA replication and transcription (107). Mitochondrial quality control at the organelle level ensures flexibility at the metabolic level, thus enabling tumor cells to adapt to targeted therapy and the tumor microenvironment and ultimately support their growth, survival, and migration (21). Studies have shown that the overexpression of c-Myc in hepatocytes promotes mitochondrial production of MAMs (endoplasmic reticulum-associated mitochondrial membrane), which induce the release of Ca^2+^ from the endoplasmic reticulum into mitochondria and oxidative stress and inhibit oxidative phosphorylation in hepatocytes in organoid containers. These events promote the initiation of hepatocellular carcinogenesis (108).

#### 3.2.1 Mitochondrial Fission

##### 3.2.1.1 Effect of Mitochondrial Fission on Proliferation of HCC Cells

Studies of the effects of mitochondrial division on the regulation of cell proliferation have (86, 87, 109) suggested that tumor cell mitochondrial dynamics affect proliferation by influencing the cell cycle. Imaging studies of cultured cells have indicated that mitochondrial morphology undergoes stereotypical changes during progression through the cell cycle (109–112). The two most apparent features are tubulation of the mitochondrial network at the G1/S transition and extensive fragmentation during mitosis. The elongated, “hyperfused” network at G1/S is associated with increased ATP production and affects the entry of cells into S phase by controlling the levels of cyclin E (111), whereas extensive fragmentation ensures an equal distribution of mitochondria to the daughter cells. Moreover, the CDK1/cyclin B-mediated phosphorylation of Drp1 at Ser616 facilitates daughter cells to accept part of the mitochondrial network, which is essential for proper progression of cell division (109).

Furthermore, Drp1 is involved in the G1/S transition in HCC cells. Overexpression of Drp1 induces mitochondrial fission, which can be inhibited by p53 (by activating the ROS-dependent Akt/MDM2 pathway) and increased activity of NF-κB (which induces cyclin D1 and E1 expression) to promote the proliferation of HCC cells (113). Inhibition of Drp1 phosphorylation suppresses mitochondrial fission, thus inhibiting cell proliferation, migration, invasion, and epithelial-mesenchymal transition (114).

Mitochondrial fission process protein 1 (MTP18) participates in mitochondrial fission by regulating the division of mitochondrial membranes. However, there limited studies have explored the function of MTP18 in mitochondrial division. Studies have suggested that the overexpression of MTP18 increases mitochondrial fragmentation and promotes HCC cell growth both *in vitro* and *in vivo* by participating in G1/S transition and inhibiting cellular apoptosis. Midiv1, an inhibitor of mitochondrial division, inhibits the growth of HCC cells (115).

##### 3.2.1.2 Impact of Mitochondrial Fission on Invasion and Metastasis in HCC

Mitochondrial fission affects the invasiveness of HCC, and increased expression of Drp1 at the mRNA and protein levels in distant metastatic tissues does not improve disease-free survival in patients with HCC. Moreover, overexpression and knockdown of Drp1 in an orthotopic liver cancer model suggested that Drp1 increases intrahepatic lung metastasis and that Drp1-induced mitochondrial fission regulates reprogramming of the adhesion complex and lamellar pseudopodia and promotes invasion in HCC by Ca^2+^/calmodulin-dependent protein kinase II. Phosphorylation of downstream extracellular signal-regulated kinase 1/2 and focal-adhesion kinase eventually promotes liver cancer invasion. Additionally, increased ATP production (by promoting Ca^2+^ influx) can repress the inhibitory effect of mitochondrial fission on cell migration, suggesting that mitochondrial division can affect invasion and metastasis in HCC by regulating mitochondrial energy production (116).

Studies of other mitochondrial fission factors have revealed high FIS1 expression in HCC metastases and showed that this protein promotes HCC invasion and migration by inducing epithelial-mesenchymal transition (117). Increased expression of MTP18 also increases the invasion of HCC cells, whereas its decreased expression inhibits invasion by suppressing epithelial-mesenchymal transition and downregulating the expression of matrix metallopeptidase 9. Furthermore, knockout of *FIS1* inhibited the migration of HCC cells, although MTP18 promoted this migration *via* mitochondrial fission (115).

##### 3.2.1.3 Impact of Mitochondrial Fission on Apoptosis in HCC

Studies suggest that Drp1-induced increased mitochondrial division enhances the survival of HCC cells by promoting mitophagy and inhibiting mitochondria-dependent apoptosis. Specifically, Drp1-mediated mitochondrial fission increases mtROS levels, which can increase NF-κB activity and inhibit TP53. Therefore, mitochondrial fission increases the viability of HCC *via* crosstalk between the two pathways (118).

However, as a regulator of mitochondrial morphology, FIS1 plays a major role in the response to stress. For example, under high-energy fructose-palmitate culture conditions, high expression of FIS1 leads to steatosis in HepG2 cells as a link in the mitochondrial stress response and induces mitochondrial fission. The mitochondria can then produce sufficient ROS to damage themselves. Other changes in the mitochondria include dissipation of the membrane potential, low expression of ETC molecules, decreased mitochondrial biosynthesis (PGC-1α), and increased caspase 3-mediated apoptosis (119). Furthermore, the addition of silver nanoparticles reduces the expression of FIS1, fragments the mitochondrial morphology (by increasing p-Drp1 levels), decreases mitochondrial biogenesis, and transiently enhances antioxidant capacity and improves mitochondrial pathway-mediated apoptosis (120).

However, some studies have shown that treatment with suberoylanilide hydroxamic acid (SAHA), an acetylase inhibitor, can significantly induce apoptosis in HCC cells through mechanisms other than the overexpression of Bcl-2. Moreover, SAHA significantly reduces the expression of FIS1 and translocation of Drp1 to the mitochondria, which inhibits mitochondrial fission. However, downregulation of the expression of proteins involved in mitochondrial division and changes in the mitochondrial morphology are not involved in SAHA-induced apoptosis in HCC cells (121).

##### 3.2.1.4 Impact of Mitochondrial Fission on Immune Regulation of HCC

Few studies focus on the impact of mitochondrial quality control of immune regulation of HCC. Bao et al. have shown that Drp1-mediated mitochondrial fission in HCC cells can induce cytoplasmic mtDNA, and promote the secretion of CCL2 which augment tumor-associated macrophages recruitment and M2 polarization eventually promote HCC cell proliferation (122). Mitochondrial quality control changes in tumor microenvironment of hepatocellular carcinoma can also affect the progression of hepatocellular carcinoma. Zheng et al. showed that Drp1-mediated mitochondrial fragmentation promoted the increase of caspase3 levels in NK cells, which augmented the apoptosis of NK cells, and objectively caused the immune escape of HCC (123).

#### 3.2.2 Mitochondrial Fusion

##### 3.2.2.1 Effect of Mitochondrial Fusion on HCC Cell Growth and Proliferation

Wang et al. found that the overexpression of MFN2 inhibits proliferation of hepatoma cells by arresting cells in S phase, and mitochondrial calcium overload caused by mitochondrial fusion mediates an increase in the expression of proteins related to the BAX apoptotic pathway (124, 125). Analysis of HCC and the surrounding tissues showed that reduced expression of MFN2 is negatively associated with the overall survival of patients with HCC (126, 127). Moreover, in HBV-related HCC, high expression of HBX induces ubiquitination and degradation of MFN2 (46), and low expression of MFN2 negatively affects the prognosis of patients with HBV-related liver cancer (128).

Tissue-specific deletion of Opa1 suggests the importance of Opa1-mediated mitochondrial fusion in the expression of ETC and cell oxidative phosphorylation metabolism (129). Compared with its expression in normal tissues, Opa1 shows low expression in HCC tissues. By reducing the expression of Opa1, Meng et al. found that this protein mediates mitochondrial fusion, promotes cell oxidative phosphorylation, and functionally supports the growth of subcutaneous HCC in mice (130).

##### 3.2.2.2 Impact of Mitochondrial Fusion on Invasion and Metastasis of HCC

Zhang et al. (131) found that MFN1 promotes mitochondrial fusion but inhibits the proliferation, invasion, and migration of HCC cells both *in vivo* and *in vitro* and that patients with HCC with high levels of MFN1 have prolonged disease-free and overall survival. Furthermore, MFN1-induced mitochondrial fusion inhibits aerobic glycolysis and a shift to oxidative phosphorylation, which induces an increase in E-cadherin expression and decrease in N-cadherin, vimentin, and SNAIL expression to eventually suppress the invasion of HCC cells.

##### 3.2.2.3 Impact of Mitochondrial Fusion on Chemoresistance in HCC

A study suggested that downregulation of Opa1 expression during treatment with sorafenib induced mitochondrial fragmentation and the release of cytochrome C to mediate apoptosis. In contrast, inhibition of Opa1 expression sensitized HCC cells to sorafenib (132).

##### 3.2.2.4 Impact of Mitochondrial Fusion on Metabolism in HCC

Most studies found that mitochondrial fusion could promote the conversion of cells metabolism from aerobic glycolysis to oxidative phosphorylation. Zhang et al. found that overexpression of MFN1 induced mitochondrial fusion in HCC can inhibit the expression of enzymes involved in aerobic glycolysis, further inhibiting cell proliferation and invasion (131). While Li et al. pointed out that MFN1 and Opa1 cooperate for prolonging mitochondria fusion, promoting oxygen consumption rate and ATP content, augment HCC proliferation (130). Li et al. found that, after the mitochondrial fusion induced by DRP1 S637 phosphorylation, the amount and width of the mitochondrial cristae were increased, assembly of the respiratory chain complex I-IV were increased, and glycolysis mediated by the NAD/SIRT1 pathway was inhibited, ultimately promoting HCC cell survival under starvation (133).

#### 3.2.3 Mitochondrial Biogenesis

##### 3.2.3.1 Effect of Mitochondrial Biogenesis on Proliferation of HCC Cells

Under hypoxic conditions, HCC and other liver cancer cell lines show increased mitochondrial biogenesis and proliferation by upregulating the expression of PGC-1α. To achieve this effect, HMGB1 translocated from the nucleus to the cytoplasm binds to cytoplasmic Toll-like receptor-9; this complex activates p38 to subsequently phosphorylate PGC-1α (134). Additionally, the p53 pathway can reduce the expression of PGC-1α, which suppresses mitochondrial biogenesis and oxidative phosphorylation by inhibiting the expression of downstream TFAM and p53R2, thus eventually decreasing the proliferation of liver cancer cells (135).

Studies of hepatocytes suggested that increased expression of Myc promotes the transcription of genes involved in biosynthesis of the mitochondrial membrane, matrix, and ribosomes; carboxylic acid metabolism; electron transporter activity; oxidative phosphorylation; and mtDNA replication by directly upregulating the expression of transcription factors, such as TFAM. This enhanced transcriptional activity increases the mitochondrial mass, which indirectly promotes the growth and proliferation of hepatocytes (21, 136).

##### 3.2.3.2 Impact of Mitochondrial Biogenesis on Invasion and Metastasis of HCC

The influence of PGC-1α-induced mitochondrial biogenesis on the invasiveness of HCC cells remains controversial. A clinical study showed that low expression of PGC-1α in HCC is related to more frequent vascular invasion. *In vitro* experiments and analysis of *in vivo* mouse models showed that PGC-1α inhibits the migration and invasion of HCC cells. Specifically, PGC-1α inhibits aerobic glycolysis by regulating the peroxisome proliferation-activated receptor-γ-dependent WNT/β-catenin/PDK1 axis, thereby inhibiting metastasis of HCC (137). NPAS2 inhibits mitochondrial oxidative phosphorylation and mitochondrial biogenesis by inhibiting the expression of PGC-1α. This inhibitory effect is synergistic with hypoxia-inducible factor-1α–mediated aerobic glycolysis in tumor cells to promote the proliferation and metastasis of HCC cells through the energy supply (138). However, another study showed that SIRT1, as part of the SIRT1/PGC-1α axis, promotes the metastasis of HCC by increasing PGC-1α-mediated mitochondrial biogenesis; a corresponding reduction in the mitochondrial mass and oxidized phosphoric acid caused by low expression of SIRT1 in liver cancer tissues inhibits invasion and metastasis (139). Therefore, a higher level of energy metabolism increases cell migration, and the mitochondrial mass and level of cellular glycolysis together affect invasion by HCC.

##### 3.2.3.3 Impact of Mitochondria Biogenesis on Chemoresistance in HCC

Further, tigecycline has been shown to inhibit the translation of mitochondrial respiratory chain complexes, enhance mitochondrial oxidative stress, and reduce resistance to cisplatin in liver cancer (140). Moreover, in cells resistant to doxorubicin and sorafenib, inhibition of TFAM, an essential factor for mitochondrial synthesis, reduces drug resistance (141).

##### 3.2.3.4 Impact of Mitochondria Biogenesis on Metabolism in HCC

PGC-1α can enhance oxidative phosphorylation, mitochondrial biogenesis and oxygen consumption rate in cancer cells (142). Under hypoxia conditions, HCC undergo changes in their bioenergetic profile to favor mitochondrial respiration by activating the PGC-1α (134). The mitochondrial respiratory capacity and proliferation was diminished when PGC-1α was suppressed in hypoxic HCC cells (143). Activation of PGC-1α was also found to promote cell growth by facilitating mitochondrial and fatty acid metabolism in liver cancer cells (144).

#### 3.2.4 Mitophagy

##### 3.2.4.1 Impact of Mitochondrial Autophagy on the Occurrence of HCC

A dysfunction in mitophagy causes tumorigenesis (145). For example, loss of mitophagy can lead to tumor formation in the liver through oxidative stress and DNA damage (146). In contrast, FUNDC1 enhances mitophagy to reduce hepatocyte inflammation and the inflammatory response to inhibit the occurrence of HCC in diethylnitrosamine-induced mouse models (147). The thyroid hormone T3 mediates ubiquitination of mitochondrial HBX in HBV-infected cells through PINK1 to simultaneously promote mitophagy and mitochondrial biogenesis, inhibit the production of mitochondrial ROS, and reduce the tumor-promoting effect of HBV (82).

##### 3.2.4.2 Impact of Mitophagy on Invasion and Metastasis in HCC

The regulation of mitophagy, *via* mechanisms other than PINK1/Parkin, can inhibit the migration of tumor cells. For example, ubiquitination of Yap, but not Parkin, reduces mitophagy by inhibiting Bnip3 to ensure the supply of ATP required for cellular maintenance. Intracellular SERCA activity and calcium homeostasis can effectively block the activation of calmodulin-dependent protein kinase II, prevent the calmodulin-dependent protein kinase II/cofilin pathway from degrading F-actin, and maintain the lamellipodium to promote the metastasis of HCC (148).

##### 3.2.4.3 Effect of Mitophagy on Chemoresistance and Resistance to Apoptosis in HCC

Studies of chemoresistance revealed that chemotherapy-induced activation of Drp1 induces mitochondrial autophagy. However, the mitochondrial division inhibitor Mdivi-1 promotes the release of cytochrome C from the mitochondria into the cytoplasm by reducing the mitochondrial membrane potential and increasing the sensitivity of HCC cells to cisplatin. These findings indicate that mitophagy increases chemoresistance in HCC. Further, inhibition of Drp1 and mitophagy can participate in downregulating the expression of Bcl-XL and upregulating the expression of Bax to enhance apoptosis, which ultimately increases the lethality of platinum-based drugs (149).

The regulation of mitophagy by the PINK1/Parkin pathway is observed in HBV-related HCC. HBV promotes cell survival and virus persistence by disrupting mitochondrial fission. Kim et al. found that HBV could induce translocation of Drp1 and Parkin to the mitochondria to mediate the degradation of MFN-2, thereby promoting mitophagy, inhibiting the mitochondrial apoptotic pathway, and maintaining tumor cell survival (46). Ketoconazole inhibits the growth of HCC cells by inhibiting prostaglandin-endoperoxide synthase 2, which activates the accumulation of PINK1 on the mitochondrial membrane and induces HCC cell apoptosis (150, 151).

A lack of mitophagy not only causes HCC but also reduces chemoresistance in HCC. It has been reported that α-TOS, a vitamin E derivative, can cause mitochondrial damage and trigger mitophagy. However, defects in the denitrosylating enzyme *S*-nitrosoglutathione reductase or Parkin reduce the ability of cells to specifically eliminate mitochondria damaged by α-TOS and decrease chemoresistance in HCC cells (152). In contrast, Wang et al. found that *Cepharanthine* hydrochloride increased mitochondrial fission and autophagy through the AMPK pathway, thereby increasing the chemoresistance of liver cancer cells. However, inhibition of mitophagy by 3-methyladenine *in vivo* and *in vitro* reduced the tumor volume, indicating improvement in the antitumor activity of *Cepharanthine* hydrochloride; thus, the inhibition of mitophagy suppresses chemoresistance (153). Increased autophagy also enhances tumor chemoresistance. For example, in HCC cells induced by melatonin, the sensitivity to sorafenib is related to the production of ROS and mitophagy. Melatonin may promote the expression of Parkin and its translocation to the mitochondrial membrane to promote mitophagy (154). A previous study by our group also showed that mitophagy induced by PINK1/Parkin influences the resistance to cisplatin, as it effectively degrades mitochondria damaged by cisplatin in HCC cells and maintains cell survival (26).

## 4 Summary and Prospects

The therapy strategy targeting mitochondrial quality control in HCC is still in the basic research stage. Mdivi-1 (mitochondrial division inhibitor 1) is a kind of selective Drp1 inhibitor. Ma et al. found that Mdivi-1 could inhibit mitophagy induced by cisplatin and initiate mitochondria pathway apoptosis, and then enhance the sensitivity of HCC to cisplatin (149). Mdivi-1 can also reduce the levels of oxidative phosphorylation and weaken the stemness in liver cancer stem cells by inhibiting mitochondrial fission (155). Lin et al. found that under hypoxic conditions, Mdivi-1 inhibits mitophagy by inhibiting the expression of BNIP3 and LC3B, up-regulating p62 and promote mitochondrial Bax accumulation, eventually stimulating mitochondrial-dependent apoptosis in HCC (76). Several other drugs like Dynasore and P110, which can target mitochondrial dynamin have been found, but few of them have been reported in the treatment of HCC, these drugs are likely to be promising therapy strategies in HCC.

In summary, mitochondrial quality control plays an important role in the occurrence, progression, and treatment of HCC. Studies have mainly focused on mitochondrial quality control at the organelle level. Mitochondrial quality control at the molecular and organelle levels can regulate bioenergy metabolism in mitochondria, control cell death, and alter mitochondrial retrograde signaling to maintain homeostasis in HCC cells under stress conditions (hypoxia and chemotherapy). However, research on mitochondrial quality control at the molecular level is primarily limited to the diagnosis, curative effect, and prognosis of patients with HCC. Studies of the impact of mitochondrial quality control at the molecular and cellular levels on the biological behavior of HCC are required.

Future research should focus on the role and mechanism of interaction between different levels of mitochondrial quality control in the regulation of the occurrence, progression, and resistance of HCC. Continuous improvements in precision medicine will clarify the process of mitochondrial quality control and role of key molecules in the precision treatment of HCC. Individual evaluation and regulation of mitochondrial quality control in HCC tissues in patients undergoing different treatments will aid in individualized treatment.

## Author Contributions

JB and DZ wrote the first draft. JS, YW, WY, and HQ provided the organization and framework of the article. JS and RC provided critical revisions. All authors contributed to the article and approved the submitted version.

## Funding

This work was supported by grants from the National Key R&D Program of China (Grant #2018YFC1311600), Natural Science Foundation of China (81902484), China Postdoctoral Science Foundation (2020M670864), Medical and Health Talents Project of Jilin Province (2019SCZT003, 2019SCZT035, 2020SCZT039), and Youth Support Project of Jilin Association for Science and Technology (202028).

## Conflict of Interest

The authors declare that the research was conducted in the absence of any commercial or financial relationships that could be construed as a potential conflict of interest.

## Publisher’s Note

All claims expressed in this article are solely those of the authors and do not necessarily represent those of their affiliated organizations, or those of the publisher, the editors and the reviewers. Any product that may be evaluated in this article, or claim that may be made by its manufacturer, is not guaranteed or endorsed by the publisher.
